# Abnormal circadian rhythm of urinary sodium excretion correlates closely with hypertension and target organ damage in Chinese patients with CKD

**DOI:** 10.7150/ijms.42875

**Published:** 2020-02-24

**Authors:** Jun Zhang, Jialing Rao, Man Liu, Wenying Zhou, Yuanqing Li, Jianhao Wu, Hui Peng, Tanqi Lou

**Affiliations:** 1 Division of Nephrology, Department of medicine, Third Affiliated Hospital of Sun Yat-Sen University, Guangzhou, Guangdong 510630, China.; 2 Division of Gastroenterology and Hepatology, the First Affiliated Hospital of Sun Yat-sen University, Guangzhou, Guangdong, China.; 3 Department of Laboratory medicine, Third Affiliated Hospital of Sun Yat-Sen University, Guangzhou, Guangdong 510630, China.

**Keywords:** circadian rhythm, urinary sodium excretion, hypertension, target-organ damage.

## Abstract

Whether the abnormal circadian rhythm of urinary sodium excretion is associated with hypertension in chronic kidney disease (CKD) is poorly understood. In this study, we assessed the relationship between the circadian rhythm of urinary sodium excretion and hypertension. Urinary samples were collected during both the day (07:00 to 22:00) and night (22:00 to 07:00) to estimate night/day urinary sodium excretion ratios. Blood pressure (BP) and clinical data were also measured. A total of 1,099 Chinese CKD patients were recruited, 308 patients were excluded, and 791 patients were final enrolled in this study. Among them, 291 patients were normotensive and 500 were hypertensive CKD patients. A 1:1 propensity score matching (PSM) analysis was performed with age and estimated glomerular filtration rate (eGFR) matched between 190 normotensive and hypertensive patients. In the full cohort and PSM cohort, multivariate regression analysis showed that the night/day urinary sodium excretion ratio was an independent risk factor for clinical hypertension, whereas 24 h urinary sodium excretion, diurnal and nocturnal urinary sodium excretion were not. When the night/day urinary sodium excretion ratios were further divided into tertiles (tertile 1 < 0.47, tertile 2, 0.47-0.84 and tertile 3 > 0.84), multivariate analysis showed that tertile 3 was independently associated with hypertension in the full and PSM cohorts. In addition, tertile 3 was also independently associated with eGFR ≤ 60 mL/min/1.73 m^2^ and left ventricular hypertrophy. These data suggested that an abnormal circadian rhythm of urinary sodium excretion was independently associated with hypertension and target-organ damage. Individualized salt intake and therapeutic strategies should be used to normalize the natriuretic dipping profile in CKD patients.

## Introduction

Chronic kidney disease (CKD) is a worldwide public health problem, with high economic costs and poor outcomes. Hypertension is the most frequent complication of CKD, and is considered both a cause and a consequence of it 1. Hypertension is also a risk factor for the progression of kidney disease and cardiovascular (CV)-associated mortality 2. A large proportion of patients with CKD have a “salt-sensitive” blood pressure (BP) phenotype because their renal sodium excretory capacity is reduced 3. Although some studies have shown a positive relationship in the CKD population between BP and 24 h urinary sodium excretion, which is considered the gold standard measurement relating salt intake and BP 4, 5, whether high dietary sodium affects the initiation or progression of CKD is still unclear. Some 6, 7, but not all 8-11 studies of patients with CKD have shown that high dietary sodium intake may increase the risk of CKD progression and reduce long-term survival. However, there have been several publications reporting negative results 12, 13 in the general population or in young people 14 regarding the association between 24 h urinary sodium excretion and hypertension.

Thus, there is still no firm data showing that 24 h urinary sodium excretion is associated with hypertension, especially with target-organ damage (TOD), CKD progression and long-term survival. A previous study measured urinary sodium excretion separately during the day and night and found that nocturnal BP and BP dipping were closely associated with the daytime urinary sodium and day/night urinary sodium excretion ratio in African subjects. In this study, the authors divided the day/night urinary sodium excretion into tertiles and found a similar level of 24 h urinary sodium excretion but different BP among the tertiles in their population 15. Therefore, more analysis employing stratification will be required to investigate these relationships.

In the CKD population, renal function deteriorates causing a decline in diurnal urinary sodium excretion, and impaired diurnal natriuresis increases pressure natriuresis, nocturnal BP, and nocturnal urinary sodium excretion to compensate for the diminished diurnal natriuresis 16, 17. Consequently, the circadian rhythm in sodium excretion may change in these patients. Because most studies have not measured urinary sodium excretion separately during the day and night in the CKD population, they also have not analyzed the association between circadian changes in sodium excretion and BP or TOD. Therefore, we investigated the relationship between diurnal, nocturnal and 24 h urinary sodium excretion and the night/day urinary sodium excretion ratio, BP and TOD (in this study, left ventricular hypertrophy, impaired renal function and abnormal carotid intima-media thickness) in a Chinese CKD population.

## Methods

### Study population

This study was approved by the Ethics Committee of the Third Affiliated Hospital of Sun Yat-sen University, Guangzhou, Guangdong, China. Written informed consent was obtained from all the patients before their enrollment. We recruited 1,099 consecutive patients from the Third Affiliated Hospital of Sun Yat-sen University between July 2010 and December 2019. The inclusion criteria were: aged 14-75 years and diagnosed with CKD 18. The exclusion criteria were: other causes resulting in secondary hypertension such as renovascular hypertension, Cushing's syndrome or aldosteronism; sustained BP higher than 200/120 mmHg; took an anti-hypertensive medication (before BP data and urinary sodium were collected, short-acting amlodipine was used according to the patient's condition) or diuretic drug in the previous month; treatment with corticosteroids or hormones; acute changes in the estimated glomerular filtration rate (eGFR) > 30% in the previous 3 months; maintenance dialysis; kidney transplantation; history of drug or alcohol abuse; employment involving night work or shiftwork; acquired immunodeficiency syndrome; inability to communicate or comply with all the study requirements; pregnancy; and refusal to provide informed consent.

**Figure 1:**
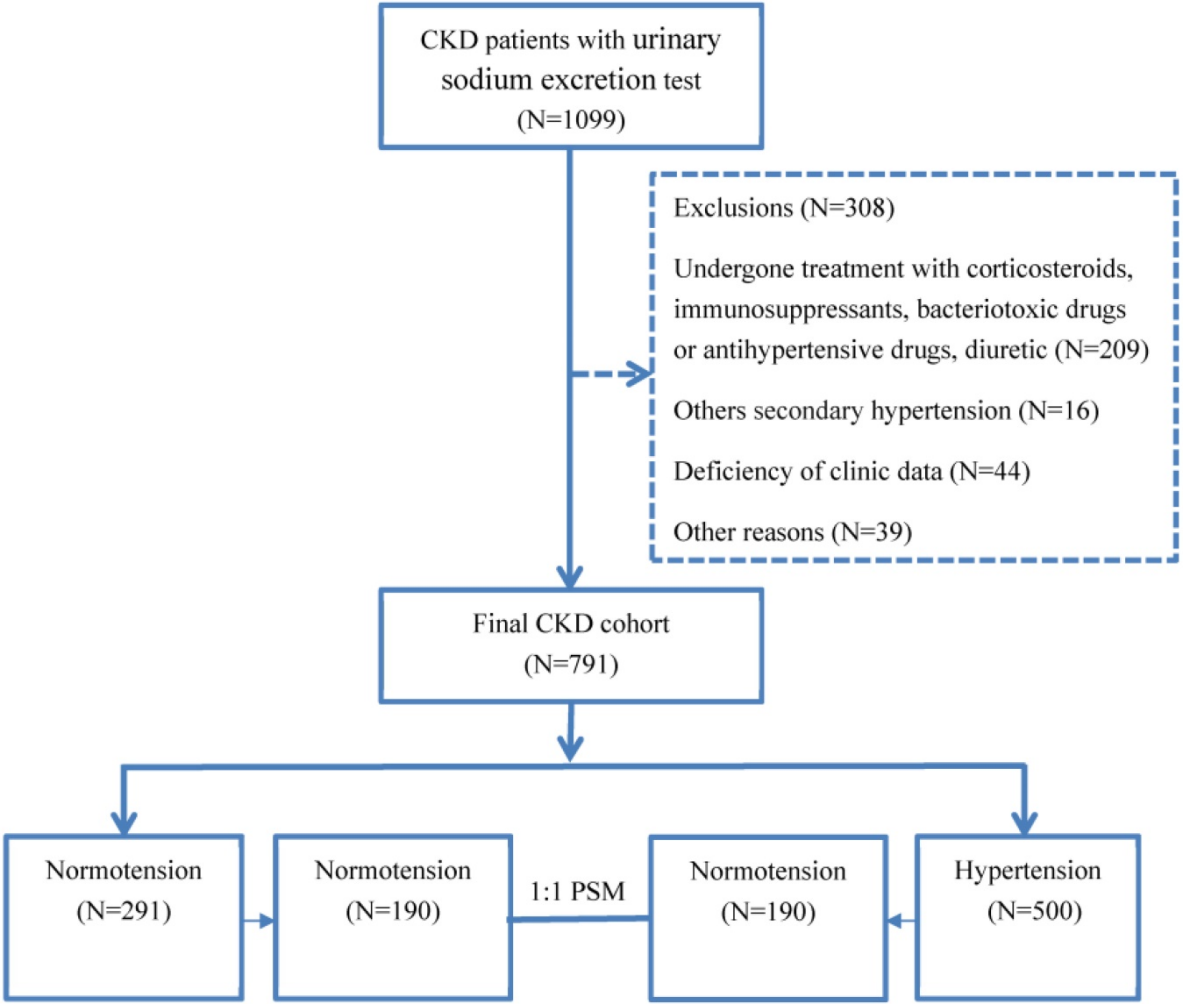
Patient enrollment flow chart.

A total of 308 patients were excluded for the following reasons (Fig. 1). In total, 791 CKD patients were enrolled in the study. The causes of CKD were chronic glomerulonephritis in 481 patients (60.81%), diabetic nephropathy in 122 patients (15.4%), interstitial nephritis in 44 patients (12.52%), obstructive nephropathy in 51 patients (3.53%), and other causes in 93 patients (7.54%; Fig. 1). Using the propensity score matching (PSM) extension program, 380 CKD patients were assigned to the hypertensive group or the normotensive group in 1:1 PSM (Fig. 1).

### Clinical data collection

BP measurements were made in a quiet environment using a mercury sphygmomanometer, with the patient in a sitting position after 5 min of rest. The details have been reported previously 19, 20. Data were collected at the initial study visit, and included demographic information, laboratory data (hemoglobin, albumin, calcium, phosphorus, intact parathyroid hormone [iPTH], fasting blood glucose, serum cholesterol, triglycerides, high-density lipoprotein-cholesterol [HDL_C], low-density lipoprotein-cholesterol [LDL_C], homocysteine, uric acid, serum creatinine [SCr], and blood urea nitrogen [BUN]), and any current treatment. The laboratory data were measured with a 7180 Biochemical Automatic Analyzer (Hitachi, Tokyo, Japan). We collected urine samples from 07:00 to 22:00 (diurnal) when the patients were awake and between 22:00 to 07:00 (nocturnal) when the patients were in bed. The patients were asked to void their bladders at 07:00 and 22:00 to ensure valid results. The diurnal and nocturnal urinary sodium levels, the 24 h urinary protein levels, and 24 h urinary sodium excretion were measured. Proteinuria was assessed by immune turbidimetry. The concentration of sodium in the urine was measured with flame spectrophotometry.

### Ultrasonographic assessment

The cardiac structure and carotid intima-media thickness (cIMT) were assessed as previously described 21. The left ventricular mass index (LVMI) was obtained by calculating the left ventricular mass to height^2.7^ 22, and patients with LVMI of 49 g/m^2.7^ (man) or 45 g/m^2.7^ (woman) were diagnosed with left ventricular hypertrophy (LVH) 23. The cIMT was assessed by two trained investigators before the commencement of the study.

### Definitions

Systolic blood pressure (SBP) < 140 mmHg and diastolic blood pressure (DBP) < 90 mmHg were defined as “normotension,” and other values were defined as 'hypertension'. CKD was defined in accordance with the KDIGO 2012 Clinical Practice Guidelines 18. eGFR levels were calculated with the 2009 CKD-EPI creatinine equation 24. Diabetes mellitus (DM) was defined as the need for antidiabetic drugs or meeting the diagnostic criteria according to the American Diabetes Association's Standards of Medical Care in Diabetes 25. TOD was defined as follows: first, left ventricular hypertrophy (LVH) was diagnosed as LVMI > 49 g/m^2^ (male) or > 45 g/m^2^ (female); second, with respect to large-vessel disease, cIMT > 1 mm was regarded as abnormal 26; third, impaired renal function was defined as eGFR < 60 mL/min/1.73 m^2^ 18. The circadian rhythm of urinary sodium excretion was determined by the night/day urinary sodium excretion ratio.

### Statistical analyses

Descriptive statistics are presented as means ± standard deviations for continuous variables and as medians and interquartile ranges for nonparametric variables. Frequencies and percentages were used for categorical variables. The differences between qualitative variables were assessed with the χ^2^ test or Fisher's exact test. Comparisons of continuous variables between groups were evaluated with Student's *t-*test or ANOVA; discontinuous variables were evaluated with nonparametric tests (the Kruskal-Wallis *H* test for several independent samples and the Mann-Whitney *U* test for two independent samples). To reduce the selection bias conferred by potential confounding factors, a PSM analysis was performed with age and eGFR matched between the normotensive and hypertensive groups. The logit of the propensity score was nearest-neighbor matched in a 1:1 manner, with a caliper of 0.02 with no replacements. Multivariable regression analyses were used to model the relationships between hypertension and urinary sodium excretion and the night/day urinary sodium excretion ratio. The entire study population was then divided into three groups according to the tertiles of the night/day urinary sodium excretion ratio, as an independent variable: tertile 1 (night/day urinary sodium excretion ratio < 0.47), tertile 2 (night/day urinary sodium excretion ratio 0.47-0.84), and tertile 3 (night/day urinary sodium excretion ratio > 0.84). The parameters of these three subgroups were compared. We used univariate and multivariate analyses to explore the relationships between hypertension, TOD, and the tertiles of the night/day urinary sodium excretion ratio. All values were two-tailed, and *P* < 0.05 was considered to indicate statistical significance. All data were analyzed with IBM SPSS Statistics, version 25.0 for Windows (IBM, Armonk, NY, USA).

## Results

### Demographic and clinical characteristics of the study population

The mean age of the full cohort was 44.5 years and 59.5% of the patients were male. The prevalence of current smokers and alcohol abusers in the full cohort was 19.7% and 10.6%, respectively, and 17.4% patients were diagnosed with DM (Table 1). Among the full cohort, patients with hypertension were older, with a higher prevalence of smokers, DM, LVH, eGFR < 60 mL/min/1.73 m2, and abnormal cIMT than the normotensive patients, and higher mean BMI, fasting glucose, serum phosphate, triglyceride, proteinuria, serum Scr, uric acid, serum cystatin C, nighttime urinary sodium excretion, night/day urinary sodium excretion ratio, LVMI, and cIMT. The hypertensive patients also had lower hemoglobin, total calcium, eGFR, cholesterol, LDL-C, HDL-C, and diurnal urinary sodium excretion. (P < 0.05; Table 1). There was no difference in 24 h urinary sodium excretion between the two groups.

In the PSM analysis that matched age and eGFR, 190 normotensive patients were pair-matched with 190 hypertensive patients in the PSM cohort. We found no differences in the demographic or clinical characteristics of the two groups, in terms of their age, sex, current smoking status, alcohol intake, hemoglobin, albumin, serum Scr, eGFR, cholesterol, LDL_C, HDL_C, uric acid, 24 h urinary sodium excretion, or diurnal urinary sodium excretion (*P* > 0.05; Table 1). However, the hypertensive group included greater proportions of patients with DM, LVH, and abnormal cIMT, higher levels of fasting glucose, triglyceride, proteinuria, LVMI, cIMT, and nocturnal urinary sodium excretion, and higher night/day urinary sodium excretion ratios than the normotensive patients (*P* < 0.05; Table 1).

### Characteristics of urinary sodium excretion in CKD patients with different BP and eGFR

**Figure 2:**
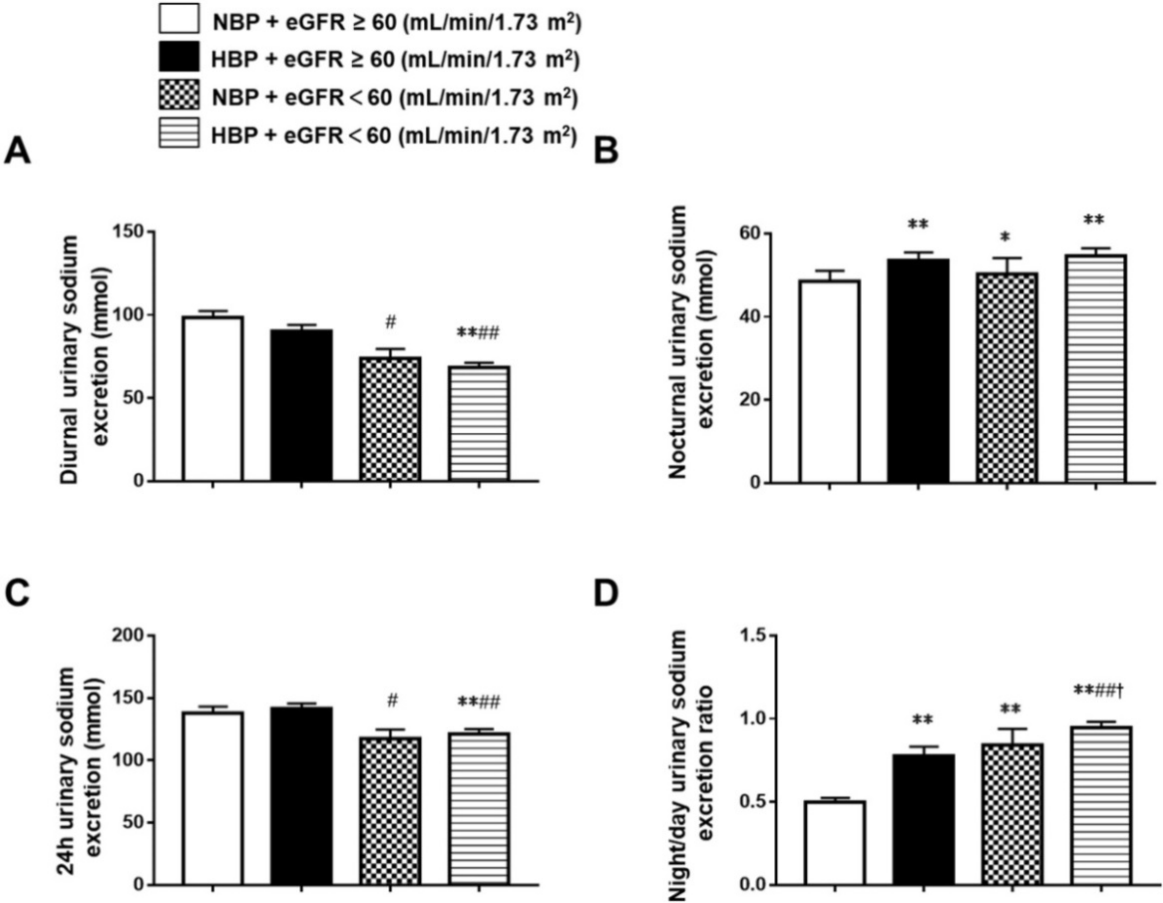
Comparison of diurnal, nocturnal, 24 h urinary sodium excretion and the night/day urinary sodium excretion ratio in Chinese CKD patients with different blood pressure and eGFR status. ^*^P < 0.05, ^**^P < 0.01 when compared with the NBP and eGFR ≥ 60 mL/min/1.73 m^2^ groups. ^#^*P* < 0.05, ^##^*P* < 0.01 when compared with the HBP and eGFR ≥ 60 mL/min/1.73 m^2^ groups. **^†^***P* < 0.05, **^††^***P* < 0.01 when compared with the NBP and eGFR ˂ 60 mL/min/1.73 m^2^ groups. NBP: normal blood pressure; HBP: high blood pressure; eGFR: estimated glomerular filtration rate.

To further understand the relationships between BP, eGFR, and urinary sodium excretion, we divided the whole group into the normotensive and eGFR ≥ 60 mL/min/1.73 m^2^ group (group 1, n = 245), the hypertensive and eGFR ≥ 60 mL/min/1.73 m^2^ group (group 2, n = 207), the normotensive and eGFR < 60 mL/min/1.73 m^2^ group (group 3, n = 54), and the hypertensive and eGFR < 60 mL/min/1.73 m^2^ group (group 4, n = 285). The 24 h urinary sodium excretion was significantly lower in group 4 (121.26 ± 66.61 mmol) than in group 1 (137.81 ± 85.45 mmol) and group 2 (141.04 ± 66.53 mmol). Group 3 (117.21± 55.40 mmol) also had significantly lower 24 h urinary sodium excretion than group 2. There were no differences in 24 h urinary sodium excretion between the hypertensive patients and the normotensive patients in the eGFR < 60 mL/min/1.73 m^2^ group or the eGFR ≥ 60 mL/min/1.73 m^2^ group. Diurnal urinary sodium excretion was significantly lower in group 4 (74.07 ± 40.35 mmol) and group 3 (68.55 ± 47.58 mmol) than in group 1 (98.49 ± 63.99 mmol) and group 2 (90.43 ± 53.70 mmol). There was no difference in diurnal urinary sodium excretion between the hypertensive patients and the normotensive patients in the eGFR < 60 mL/min/1.73 m^2^ group or the eGFR ≥ 60 mL/min/1.73 m^2^ group. Nocturnal urinary sodium excretion was significantly higher in group 2 (53.47 ± 29.71 mmol), group 3 (50.35 ± 27.14 mmol) and group 4 (54.73 ± 31.29 mmol) when compared to group 1 (48.60 ± 48.60 mmol). In the hypertensive group, there was no difference in nocturnal urinary sodium excretion between patients with different eGFR levels, and nocturnal urinary sodium excretion did not differ between the hypertensive and normotensive patients when eGFR < 60 mL/min/1.73 m^2^. The night/day urinary sodium excretion ratio was significantly higher in group 2 (0.58, 0.36-0.88), group 3 (0.65, 0.53-0.86), and group 4 (0.85, 0.55-1.16) than in group 1 (0.41, 0.27-0.61), and it was also significantly higher in group 4 than in the other groups. It did not differ between groups 2 and 3 (Figure 2).

### Regression analysis of the relationships between hypertension and different kinds of urinary sodium excretion

In a multivariate regression analysis of the full cohort (Table 2, model 1), hypertension was independently associated with low diurnal urinary sodium excretion, high nocturnal urinary sodium excretion, and a high night/day urinary sodium excretion ratio, but not with 24 h urinary sodium excretion. In model 2, in which the factors significantly associated with urinary sodium in model 1 were included in a multivariate regression analysis, we found that only the night/day urinary sodium excretion ratio was independently associated with hypertension (Table 2).

To adjust the selection bias conferred by potential confounding factors, a PSM analysis was performed. In model 1, we found that high nocturnal urinary sodium excretion and a high night/day urinary sodium excretion ratio were associated with hypertension. The same analysis of the whole cohort found that only the night/day urinary sodium excretion ratio was independently associated with hypertension in model 2 (Table 2).

### Baseline characteristics and the different tertiles of the night/day urinary sodium excretion ratio

To further analyze the effects of the night/day urinary sodium excretion ratio on hypertension and TOD in CKD patients, we divided them into three equally sized groups: tertile 1 (night/day urinary sodium excretion ratio < 0.47), tertile 2 (0.47-0.84), and tertile 3 (night/day urinary sodium excretion ratio > 0.84). Tertile 3 had a higher proportion with DM, LVH, and eGFR < 60 mL/min/1.73 m^2^, was older, had higher serum phosphate, serum Scr, serum cystatin C, iPTH, nocturnal urinary sodium excretion, LVMI, SBP and DBP, and had lower hemoglobin, eGFR-MDRD and diurnal urinary sodium excretion than tertile 1 and tertile 2. Tertile 3 also had higher fasting glucose, uric acid, and cIMT, and lower cholesterol, HDL_C, LDL_C, and 24 h urinary sodium excretion than tertile 1 (Table 3). Tertile 2 had a higher prevalence of DM, hypertension, abnormal cIMT, and LVH, older age, higher serum phosphate, uric acid, serum cystatin C, Scr, nocturnal urinary sodium excretion, LVMI, and cIMT, and lower hemoglobin, LDL_C, HDL_C, diurnal urinary sodium excretion, 24 h urinary sodium excretion, and eGFR than tertile 1 (Table 3).

### Regression analysis of the relationships between hypertension, TOD, and different tertiles of the night/day urinary sodium excretion ratio

In the unadjusted regression analyses, tertile 2 and tertile 3 in the full cohort and tertile 3 in the PSM cohort were significantly associated with hypertension. After adjustment for age, sex, DM, current smoking status, alcohol intake, BMI and variables significantly associated with hypertension in the univariate regression analysis, tertile 2 and tertile 3 in the full cohort and PSM cohort were all independently related to hypertension (p < 0.05; Table 4).

We then analyzed the relationship between TOD and different tertiles of the night/day urinary sodium excretion ratio. Univariate regression analysis showed that tertile 2 and tertile 3 in the full cohort were associated with lower eGFR, LVH, and abnormal cIMT. After adjustment for factors significantly associated with TOD variables in the univariate regression analysis, including age, sex, DM, current smoking status, alcohol intake, BMI, hemoglobin, albumin, calcium, phosphate, serum fasting glucose, cholesterol, triglycerides, HDL-C, LDL-C, uric acid, proteinuria and eGFR, the tertile 3 group remained independently associated with hypertension, lower eGFR, and LVH, but not with abnormal cIMT (Table 5).

## Discussion

In this study, we evaluated BP and TOD in relation to diurnal, nocturnal, and 24 h urinary sodium excretion and the night/day urinary sodium excretion ratio. The following findings have emerged. First, we demonstrated that there was no significant difference in the 24 h urinary sodium excretion rates between normotensive and hypertensive CKD patients. Second, in our cohort, patients with eGFR ≥ 60 mL/min/1.73 m2 and the normotensive groups had the lowest night/day urinary sodium excretion ratios. The hypertensive patients with eGFR < 60 mL/min/1.73 m2 had the highest night/day urinary sodium excretion ratio of all the groups. Third, multivariate logistic regression analyses of the full cohort and the PSM cohort showed that high night/day urinary sodium ratios correlated closely with hypertension. Fourth, multivariate regression analysis showed that the tertile with the highest night/day urinary sodium excretion ratio (> 0.84) was independently associated with hypertension, lower eGFR and LVH.

We observed lower 24 h urinary sodium excretion in patients in the eGFR < 60 mL/min/1.73 m2 group, which was also reported in a previous study 9. This was perhaps attributable to a reduced dietary intake of salt related to poor appetite. In addition, we observed no positive relationship between BP and 24 h urinary sodium excretion, which is inconsistent with a previous CKD study 5. Several factors may explain this discrepancy. First, the effects of confounding factors, such as age, genetics, environmental factors, different CKD stages, and comorbidities, may cause CKD patients to display different salt-sensitive BP changes. Second, the average of 24 h urinary sodium excretion was 139.51±77.06 mmol in normotensive patients and 133.71±67.63 mmol in hypertensive patients, which is a relatively lower level in our CKD cohort compared to the previous study 5. Therefore, it was difficult to demonstrate a positive relationship between 24 h urinary sodium excretion and BP levels.

In this study, we observed that the night/day urinary sodium excretion ratio correlated with hypertension. The potential mechanism has been explained by Fukuda et al. in 2010^17^. A reduced renal sodium excretory capacity has been recognized in the salt-sensitive type of hypertension and in CKD patients, and when the salt intake is higher than appropriate, a defect in the diurnal sodium excretory capacity becomes evident, and urinary sodium excretion remains relatively low, resulting in elevated nocturnal BP to compensate for the diminished natriuresis during the daytime. This increases pressure natriuresis and more sodium is excreted during the night, so the normal circadian rhythm of urinary sodium excretion is disturbed 17. If nocturnal pressure natriuresis compensates for the reduced sodium excretion from the kidneys during the day, high BP may continue during the night until sufficient excess sodium is excreted in the urine.

Kidney Disease Improving Global Outcomes (KDIGO) suggests a reduction to < 2 g/day sodium, corresponding to 5 g/day salt, for adult patients with CKD 18. These recommendations are based on low level evidence from studies with marked heterogeneity 27. The current recommendations make no distinction between CKD stage or comorbidities. Furthermore, the KDIGO does not account for salt sensitivity in their recommendations. Recommending the same salt cut-off value for the whole CKD population may not benefit all patients. The circadian rhythm of urinary sodium excretion may be a sensitive index for sodium excretory capacity and salt sensitivity, which could predict both high BP and TOD. The recommended total salt intake should be individualized, with the patient's circadian rhythm of urinary sodium excretion taken into consideration. For example, in a CKD hypertensive patient with a normal circadian rhythm of urinary sodium excretion, salt intake may not be the reason for hypertension. Therefore, strictly limiting his/her salt intake may be of no benefit to his/her BP. Recently studies revealed an association between low urinary sodium excretion and worse prognosis 28, 29. More studies are required to investigate the relationship between the circadian rhythm of urinary sodium excretion and BP.

This study had several advantages and limitations. To the best of our knowledge, this is the first study to explore the relationships between the circadian rhythm of urinary sodium excretion, hypertension, and TOD in a CKD population. Urinary sodium excretion was measured separately and provided more information regarding the significance of urinary sodium excretion during the day and night, especially night/day urinary sodium excretion ratio. The potential limitations of our study include that is was a real world observational cross-sectional study and therefore could not establish the cause-effect relationship between the circadian rhythm of urinary sodium excretion and BP. In the future, a follow-up study should investigate the relationships between the circadian rhythm of urinary sodium excretion and hypertension, TOD and the prognosis of CKD; This study did not allow us to determine the mechanism(s) by which a higher night/day sodium excretion ratio is associated with TOD; Although they do not compensate for a truly random design, the large number of subjects in our study and controlling for several potential confounders (age, GFR) with PSM and multivariate regression analyses improved the credibility of our results.

In conclusion, the results of this study clearly demonstrated that in patients with CKD, an abnormal circadian rhythm of urinary sodium excretion is closely related to hypertension and TOD. In CKD patients, the recommended salt intake should also take into account the individual's circadian rhythm of urinary sodium excretion. We suggest that in addition to 24 h urinary sodium excretion, both diurnal and nocturnal urinary sodium excretion should be measured in these patients.

## Figures and Tables

**Table 1:** Differences in demographic and clinical characteristics of ambulatory normotensive patients and hypertensive patients.

	Full cohort				PSM cohort^ɑ^			
	Total (N=791)	Normotension (N=291)	Hypertension (N=500)	*P*	Total (N=380)	Normotension (N=190)	Hypertension (N=190)	*P*
Age (years)	44.54±15.77	38.65±14.94	47.96±15.24	<0.001	42.66±15.25	42.69±15.20	42.63±15.35	0.968
Male: female ratio	471:320	163:128	308:192	0.133	230:150	109:81	121:69	0.248
Current smoker, n (%)	156 (19.7)	45 (15.5)	111 (22.2)	0.026	77 (20.3)	33 (173.4)	43 (22.6)	0.200
Alcohol intake, n (%)	84 (10.6)	24 (8.2)	60 (12.0)	0.119	36 (9.5)	13 (6.8)	23 (12.1)	0.082
Diabetes mellitus, n (%)	138 (17.4)	23 (7.9)	115(23.0)	<0.001	56 (14.7)	20 (10.5)	36 (18.9)	0.029
BMI (kg/m^2^)	23.64±4.69	22.33±3.43	23.62±3.53	<0.001	23.13±3.39	22.40±3.23	23.84±3.89	<0.001
Total calcium (mmol/L)	2.18±0.24	2.21±0.25	2.16±0.23	0.031	2.21±0.25	2.23±0.27	2.18±0.23	0.040
Serum phosphate (mmol/L)	1.42±0.46	1.29±0.32	1.49±0.51	<0.001	1.33±0.40	1.33±0.37	1.33±0.41	0.992
Serum sodium (mmol/L)	139.89±9.21	140.21±2.94	139.71±11.31	0.27	140±3.01	140.24±3.17	140.85±2.82	0.055
Hemoglobin (g/L)	112.22±29.64	123.41±24.16	105.78±30.59	0.001	119.82±28.63	118.75±26.23	120.89±30.89	0.469
Albumin (g/L)	34.39±8.17	34.26±9.13	34.47±9.57	0.742	34.47±8.33	35.31±8.42	33.63±8.17	0.051
Fasting glucose (mmol/L)	5.16±1.51	4.88±1.11	5.32±1.67	<0.001	5.19±1.57	5.01±1.24	5.36±1.83	0.031
Cholesterol (mmol/L)	5.57±2.48	5.87±2.64	5.40±2.36	0.012	5.58±2.58	5.53±2.50	6.03±2.65	0.068
Triglyceride (mmol/L)	1.96±1.68	1.76±1.36	2.08±1.83	0.012	2.02±1.68	1.77±1.29	2.27±1.98	0.004
HDL-C (mmol/L)	1.18±0.42	1.31±0.48	1.11±0.37	<0.001	1.22±0.45	1.24±0.47	1.21±0.42	0.570
LDL-C (mmol/L)	3.55±1.85	3.82±2.00	3.39±1.75	0.002	5.01±2.51	3.56±1.82	3.88±2.00	0.112
Uric acid (mmol/L)	483.53±152.10	430.41±144.26	513.54±148.31	0.254	463.24±147.02	451.02±153.47	457.39±139.67	0.107
Serum cystatin C (mg/L)	2.49±1.75	1.56±1.30	3.05±1.75	<0.001	1.98±1.52	1.89±1.48	2.07±1.57	0.268
Serum creatinine (μmol/L)	151.5 (84.0-489.3)	86.0 (64.15-143.60)	278.9 (120.0-628.7)	<0.001	107.55 (78.00-228.50)	108.10 (75.73-222.18)	106.10 (78.55-255.75)	0.759
eGFR-MDRD (mL/min/1.73m^2^)	52.74±45.00	81.87±43.49	36.22±36.74	<0.001	64.34±41.18	64.51±40.80	64.18±41.66	0.939
eGFR <60 (mL/min/1.73 m^2^)	348 (44.0)	51 (17.5)	297 (59.4)	<0.001	105 (27.6)	52 (27.4)	53 (27.9)	1.000
Proteinuria (g/d)	1.48 (0.48-3.88)	0.87 (0.26-2.8)	1.87(0.76-4.12)	<0.001	1.36 (0.42-3.44)	0.75 (0.22-2.40)	2.00 (0.75-4.42)	<0.001
24 h UNa excretion (mmol)	135.84±71.25	139.51±77.06	133.71±67.63	0.270	138.57±67.99	135.71±69.42	141.44±66.01	0.412
Diurnal UNa excretion (mmol)	84.73±53.72	94.35±57.09	79.13±50.89	<0.001	88.93±53.02	89.95±54.45	87.92±57.67	0.709
Nocturnal UNa excretion (mmol)	51.12±34.22	45.16±36.23	54.58±32.53	<0.001	49.64±28.78	45.75±25.82	53.53±31.05	0.008
Night/day UNa excretion ratio	0.77±0.63	0.58±0.46	0.88±0.68	<0.001	0.71±0.66	0.62±0.48	0.81±0.78	0.005
LVMI (g/m^2.7^)	49.66±16.14	39.69±10.10	55.00±15.24	<0.001	46.00±14.01	41.76±10.44	49.80±15.66	<0.001
LVH, n (%)	390 (49.3)	65 (22.3)	325 (65.0)	<0.001	144 (37.9)	52 (27.4)	92 (48.4)	<0.001
cIMT (mm)	0.74±0.26	0.63±0.20	0.79±0.27	<0.001	0.72±0.25	0.67±0.23	0.77±0.27	0.011
Abnormal cIMT, n (%)	190 (24)	39 (13.4)	151 (30.2)	<0.001	84 (22.1)	27 (14.2)	57 (30.0)	<0.001
SBP (mmHg)	134.31±18.40	115.58±8.29	145.22±13.12	<0.001	129.75±16.31	117.14±7.58	142.36±12.52	<0.001
DBP (mmHg)	80.22±10.66	70.35±5.02	85.98±8.67	<0.001	78.31±9.91	71.09±4.91	85.54±8.24	<0.001

PSM: propensity score matching; DBP: diastolic blood pressure; SBP: systolic blood pressure; DM: diabetes mellitus; eGFR: estimated glomerular filtration rate; HDL-C: high-density lipoprotein-cholesterol; LDL-C: low-density lipoprotein-cholesterol; iPTH: intact parathyroid hormone; cIMT: carotid intima-media thickness; LVMI: left ventricular mass index; LVH: left ventricular hypertrophy. **^ɑ^**PSM analysis was performed with age and eGFR matched between the normotensive and hypertensive groups. *P* values are for the comparison between normotensive and hypertensive patients.

**Table 2:** Multivariate logistic regression analysis of the relationship between hypertension and urinary sodium excretion in patients with CKD.

Variable	Full cohort (N=791) Multivariate regression analysis OR (95% CI)		PSM cohort (N=380) ɑ Multivariate regression analysis OR (95% CI)	
	Model 1	Model 2	Model 1	Model 2
24 h UNa excretion (per mmol)	1.000 (0.997 ‐1.003)	-	0.999 (0.994 ‐1.003)	-
Diurnal Urinary sodium excretion (per mmol)	0.996 (0.993 ‐1.000)*	1.000 (0.995 ‐1.004)	0.995 (0.989 ‐1.000)	-
Nocturnal UNa excretion (per mmol)	1.008 (1.001 ‐1.016)*	1.000 (0.993 ‐1.008)	1.009 (1.001 ‐1.017)*	1.003 (0.994 ‐1.002)
Night / day UNa excretion ratio	2.269 (1.383 ‐3.721)**	2.085 (1.147 ‐3.790)*	2.043 (1.258 ‐3.317)**	1.878 (1.105 ‐3.192)*

PSM: propensity score matching. CI: confidence interval. Urinary sodium excretion with no significant associations in model 1 was not included in model 2. Model 1: multivariate logistic regression analysis of the relationships between hypertension and 24 h urinary sodium excretion, diurnal urinary sodium excretion, nocturnal urinary sodium excretion and the day/night urinary sodium excretion ratio. Variables for the simple regression analysis of hypertension included age, sex (female = 0, male = 1), diabetes mellitus, current smoking status, alcohol intake, BMI, hemoglobin, LDL-C, calcium, phosphate, iPTH, 24 h proteinuria and eGFR (1 = eGFR ≥60 mL/min/1.73 m^2^; 2 = eGFR <60 ml/min/1.73 m^2^). Model 2 variables: age, sex (female = 0, male = 1), diabetes mellitus, current smoking status and alcohol intake; variables cited above that were significantly associated with hypertension in model 1 were also included in the multiple regression analysis. **^ɑ^**PSM analysis was performed with age and eGFR matched between the normotensive and hypertensive groups. ^*^P < 0.05, ^**^P < 0.01.

**Table 3:** Baseline characteristics of patients with CKD stratified by tertiles of the night/day urinary sodium excretion ratio.

Variable	Night / day Urinary sodium excretion ratio		
	T1 < 0.47 (n = 264)	T2 0.47-0.84 (n = 264)	T3 > 0.84 (n = 263)
Age (years)	38.62±14.82	45.35±15.53^**^	47.96±15.24^**##^
Male: female ratio	166 : 98	159 : 105	150 :114
Current smoker, n (%)	53 (20.1)	50 (18.9)	52 (19.8)
Alcohol intake, n (%)	9 (3.4)	11 (4.2)	8 (3.0)
Diabetes mellitus, n (%)	27 (10.2)	46(17.4)^*^	66(25.1)^**#^
BMI (kg/m^2^)	22.25±3.61	22.84±3.37	23.36±3.65
Total calcium (mmol/L)	2.21±0.22	2.18±0.27	2.15±0.28
Serum phosphate (mmol/L)	1319±0.33	1.42±0.44^**^	1.52±0.56^**##^
Serum sodium (mmol/L)	140.24±3.39	140.20±8.99	140.31±3.37
Hemoglobin (g/L)	124.15±28.23	112.00±28.68^**^	100.58±22.30^**##^
Albumin (g/L)	34.31±9.25	34.76±8.18	34.10±6.89
Fasting glucose (mmol/L)	4.93±1.20	5.19±1.57	5.35±1.69^*^
Cholesterol (mmol/L)	5.96±2.67	5.47±2.48^*^	5.29±2.21^**^
Triglyceride (mmol/L)	1.94±1.42	2.05±1.81	1.88±1.79
HDL-C (mmol/L)	1.25±0.45	1.16±0.43^*^	1.14±0.40^**^
LDL-C (mmol/L)	3.94±2.09	3.43±1.80^**^	3.28±1.59^**^
Uric acid (mmol/L)	451.69±137.72	493.60±148.65^**^	505.21±164.30^**^
Serum cystatin C(mg/L)	1.74±1.29	2.59±1.86^**^	3.16±1.75^**##^
Serum creatinine (μmol/L)	97.90 (68.80-185.75)	156.00 (92.10-474.75)^ **^	332.30 (122.87-688.25)^ **##^
eGFR-MDRD (mL/min/1.73m^2^)	74.61±45.69	48.27±40.69^**^	35.33±34.42^**##^
eGFR ≤ 60 (mL/min/1.73 m^2^)	61 (23.1)	116 (43.9)^ **^	170 (64.6)^ **##^
Proteinuria (g/d)	1.45 (0.38-3.63)	1.45(0.44-3.59)	1.77(0.69-4.16)
24 h UNa excretion (mmol)	149.51±78.98	131.02±61.09^**^	126.77±69.17^**^
Diurnal UNa excretion (mmol)	117.15±65.63	81.16±39.81^**^	55.88±30.39^**##^
Nocturnal UNa excretion (mmol)	32.71±17.41	49.86±24.22^**^	51.12±34.22^**##^
Night/day UNa excretion ratio	0.300±0.10	0.63±0.10^**^	1.40±0.73^**##^
LVMI (g/m^2.7^)	44.24±14.61	49.10±14.42^**^	55.28±17.28^**##^
LVH, n (%)	87 (32.9)	126 (47.7)^ **^	174 (66.2)^**##^
cIMT (mm)	0.67±0.24	0.74±0.28^*^	0.79±0.23^**##^
Abnormal cIMT, n (%)	36 (13.6)	62 (23.5)^ **^	86 (32.7)^**#^
Clinic-SBP (mmHg)	128.10±18.93	134.16±16.50^**^	140.73±17.61^**##^
Clinic-DBP (mmHg)	76.80±10.62	80.57±10.12^**^	83.31±10.25^**##^
Hypertension, n (%)	117 (44.8)	170 (63.0)^**^	212 (81.9)^**##^

DBP: diastolic blood pressure; SBP: systolic blood pressure; DM: diabetes mellitus; eGFR: estimated glomerular filtration rate; BMI: body mass index; HDL-C: high-density lipoprotein-cholesterol; LDL-C: low-density lipoprotein-cholesterol; iPTH: intact parathyroid hormone; cIMT: carotid intima-media thickness; LVMI: left ventricular mass index; LVH: left ventricular hypertrophy. ^*^P < 0.05, ^**^P < 0.01 when compared with tertile 1. ^#^*P* < 0.05, ^##^*P* < 0.01 when compared with tertile 2.

**Table 4:** Logistic regression analysis of the relationship between hypertension and different tertiles of the night/day urinary sodium excretion ratio in full and PSM cohorts of patients with CKD.

Hypertension, n (%)	Univariate regression analysis	Multivariate regression analysis
	OR (95% CI)	OR (95% CI)
**Full cohort (N=791)**		
T1, 117 (44.8)	Reference	Reference
T2, 170 (63.0)	2.092 (1.479 - 2.092)^**^	2.092 (1.479 - 2.092)^**^
T3, 212 (81.9)	5.578 (3.742 - 8.313)^**^	5.578 (3.742 - 8.313)^**^
**PSM cohort (N=380) ^ɑ^**		
T1, 58 (41.4%)	Reference	Reference
T2, 70 (49.0%)	1.356 (0.848 ‐ 2.168)	2.181 (1.210 ‐ 3.944)^*^
T3, 62 (63.9%)	2.504 (1.469 ‐ 4.270)^**^	3.700 (1.867 ‐ 7.332)^**^

PSM: propensity score matching. Variables for the multivariate regression analysis of hypertension in the full cohort included age, sex, diabetes mellitus, current smoking status, alcohol intake, BMI, hemoglobin, LDL_C, TG, total calcium, serum phosphate, fasting glucose, iPTH, 24 h proteinuria and eGFR (1 = eGFR ≥ 60 mL/min/1.73 m^2^; 2 = eGFR <60 ml/min/1.73 m^2^). Variables for the multivariate regression analysis of hypertension in the PSM cohort included age, sex, diabetes mellitus, current smoking status, alcohol intake, BMI, total calcium, serum phosphate, fasting glucose and 24 h proteinuria. **^ɑ^**PSM analysis was performed with age and eGFR matched between the normotensive and hypertensive groups. ^*^P < 0.05, ^**^P < 0.01.

**Table 5:** Logistic regression analysis of the relationship between eGFR < 60 ml/min/1.73 m^2^, LVH, abnormal cIMT and different tertiles of the night/day urinary sodium excretion ratio in patients with CKD.

Variable	Univariate regression analysis	Multivariate regression analysis
	OR (95% CI)	OR (95% CI)
**eGFR (1 = eGFR ≥ 60 mL/min/1.73 m^2^; 2 = eGFR < 60 mL/min/1.73 m^2^)**		
T1	Reference	Reference
T2	2.607 (1.782 - 3.813)^**^	1.890 (0.982 - 3.640)
T3	6.115 (4.144 - 9.023)^**^	2.675 (1.365 - 5.243)^**^
**LVH (1 = no LVH; 2 = LVH)**		
T1	Reference	Reference
T2	1.859 (1.202 - 2.873)^**^	1.356 (0.735 - 2.479)
T3	4.005 (2.568 - 6.244)^**^	2.050 (1.076 - 3.906)^*^
**cIMT (1 = cIMT≤1 mm; 2 = cIMT>1 mm)**		
T1	Reference	Reference
T2	1.961 (1.014 - 3.794)^*^	1.344 (0.572 - 3.158)
T3	3.081 (1.615 - 5.879)^**^	1.653 (0.696 - 3.922)

cIMT: carotid intima-media thickness; LVMI: left ventricular mass index; LVH: left ventricular hypertrophy. Variables for the multivariate regression analysis of LVH and abnormal cIMT included age, sex, diabetes mellitus, current smoking status, alcohol intake, BMI, hemoglobin, LDL_C, total calcium, serum phosphate, iPTH, 24 h proteinuria, eGFR (1 = eGFR ≥ 60 mL/min/1.73 m^2^; 2 = eGFR <60 mL/min/1.73 m^2^) and blood pressure (1=normotension, 2=hypertension). Variables for the multivariate regression analysis of eGFR < 60 mL/min/1.73 m^2^ included age, sex, diabetes mellitus, current smoking status, alcohol intake, BMI, hemoglobin, LDL_C, total calcium, serum phosphate, iPTH, 24 h proteinuria and blood pressure (1=normotension, 2=hypertension). ^*^P < 0.05, ^**^ < 0.01.
